# Short selling and intraday volatility: evidence from the Chinese market

**DOI:** 10.1186/s40064-015-1591-5

**Published:** 2015-12-22

**Authors:** Yongjie Zhang, Keming Liu, Dehua Shen, Wei Zhang

**Affiliations:** College of Management and Economics, Tianjin University, 300072 Tianjin, People’s Republic of China; China Center for Social Computing and Analytics, Tianjin University, 300072 Tianjin, People’s Republic of China

**Keywords:** Short selling, Intraday volatility, Relative ranking, Chinese stock market

## Abstract

The implementation of margin trading and securities lending mechanism offers us a unique circumstance to analyze the impact of short selling regulations in China. We define the addition events as the stocks are included to the designated securities list and therefore can be sold short. By focusing on the 30 trading days around the addition events, the results document statistically significant post-event increase in volatility relative to the overall market and absolute value of trading volume. Specifically, small-cap stocks experience the sharpest increase. The robustness is also performed to validate the results.

## Background

In spite of the fiercely blamed for decline in stock prices during the financial crisis, the China Securities Regulatory Commission (CSRC) approved a pilot program for margin trading and securities lending on 31 March 2010. This program allows investors to buy or sell listed securities with borrowed money or securities from stock exchange’s member securities firms by pledging collaterals to the latter. Regulators in China expect that the policy would help improve the price discovery mechanism, increase liquidity of the securities market and provide a new trading option for investors. The margin trading and securities lending program can be recognized as the short selling regulations in China. This event provides us a unique opportunity to investigate the impact of short selling mechanism on the market quality of Chinese stock market. Meanwhile, this approval conflicts with the US’s short selling regulations of July 2008, which barred naked short sales and required short sellers to borrow shares before trading. Given the special characteristic of the Chinese market, i.e., the market is dominated by individual investor, there exists price limits and the stock market is easily affected by the political events, empirically examining the impact of short selling mechanism on market quality remains of interest to financial economists and policy maker in China.

### Literature review

It is generally accepted that short selling constraints bias upward or downward the security prices (Miller 1977; Jarrow 1980; Figlewski 1981). Financial theories on the role of short sellers and their consequences of trading are mixed. On the one hand, short sellers are considered as informed traders (Boehmer et al. 2008; Diamond and Verrecchia 1987), who move the mispriced securities closer to their fundaments by actively trading. On the other hand, trade-based manipulation schemes and predatory trading strategy of short sellers move the securities prices deviated from their fundaments (Allen and Gale 1992; Brunnermeier and Pedersen 2005; Goldstein and Guembel 2008). Therefore, it is an empirical question to ask what the role of short sellers in China is. The primary motivation of this paper, is the empirically findings of this rarely-concerned question.

Taken broadly, much of the empirical work on short selling can be traced back to Miller (1977) and Diamond and Verrecchia (1987). Miller (1977) argues that short selling constraints drive pessimistic traders out of the market, enabling the prices only reflect the valuations of the more optimistic traders; while Diamond and Verrecchia (1987) focus on the speed of price adjusted to efficiency due to lacking of informative traders. Most recently, a number of studies have analyzed the effect of the US 2008 short selling regulations. Boulton and Braga-Alves (2010) find that the restrictions negatively impact various measures of liquidity, including bid-ask spreads and trading volume. Boehmer et al. (2013) find that the small-cap stocks are broadly unaffected, but large-cap stocks suffer a severe decline in market quality, as measure by price impact, bid-ask spreads and stock price volatility. Kolasinski et al. (2013) show that the short selling restrictions do not reduce informed trading activities and may result in an increase by increasing the proportion of informed short sellers to uninformed ones. Some international evidences can also be found in Clifton and Snape (2008) and Gagnon and Witmer (2014). Specifically, for the Asia—Pacific market, Chang et al. (2007) find that short selling restrictions tend to cause the individual stock overvaulted. Hamson et al. (2008) find a decrease in market quality, i.e., market liquidity become decreased and the intraday volatility increased, when the ASIC’s short selling ban is introduced. Bohl et al. (2012) also find that short selling restrictions increase stock return volatility.

The objective of this paper is on investigating the impact of short selling mechanism on Chinese stock market. On a different methodology, but closely related to our paper, Chang et al. (2014) examined the impact of short selling on Chinese market. However, most of their focuses are on the price efficiency measurements and fail to identify the temporary intraday volatility, i.e., the impact of short selling mechanism on the period from 1 month before to 1 month after the implementation is deeply ignored. Hongwei and Xin (2012) examined the price efficiency of the underlying stocks based on the Difference-in-Differences model and concluded that the overall effect of marginal trading and securities lending mechanism was insignificant. However, the Difference-in-Differences model is not suitable (see “Relative ranking methodology” for details) in China and therefore their conclusions are problematic. Besides, Zhang et al. (2015) viewed the short selling mechanism as the natural experiment for the changes of information environment and discussed impact of information environment on suitability of R^2^ and idiosyncratic volatility as proxies for firm-specific return variation.

In consequence, the rationale of this study is that using an appropriate methodology to extract the temporary impact of short selling mechanism on the market volatility. Our paper contributes to the literature in three main ways. Firstly, rather than focusing on the price efficiency, the intraday volatility is firstly examined and various calculations of volatility are performed for robustness. Secondly, we propose the relative ranking method to difference out both the firm-level and macroeconomic-level confounding factors on condition that no proper control group could be identified. This method can be employed to examine the impact of other events in stock market. Thirdly, the empirical results would provide some managerial implications for the policy maker who is interested in improving trading mechanism and boosting the development of the Chinese stock market.

## Methods

### Short selling regulations in China

On 31 March 2010, with aim of enhancing the fundamental system of capital market and perfecting the securities trading modes, the short selling regulations, i.e., margin trading and securities lending, was launched. Totally, 90 constituent stocks in the SSE 50 Index and SZSE Component Index are initialed approved to sell short. Up to 3 May 2013, the designated securities list is revised 10 times. Specifically, seven exchange-traded funds were included on 5 December 2011. Summary statistics about these revisions are reported in Table 1.

**Table 1:** List changes, addition events and deletion events

Effective date	Number of stocks on the list	Addition events	Deletion events	Stock exchanges (SSE = 1; SZSE = 0)
2010/3/31	90	90	N/A	1; 0
2010/7/1	90	5	5	1; 0
2010/7/29	90	1	1	1
2011/12/5	278	189	1	1; 0
2013/1/31	500	276	54	1; 0
2013/3/6	499	N/A	1	1
2013/3/7	498	N/A	1	0
2013/3/29	496	N/A	2	1; 0
2013/5/2	495	N/A	1	1
2013/5/3	494	N/A	1	0
Cumulated	494	561	67	N/A

This table reports the occurrence of events in which stocks on Chines stock market experienced the short selling regulations. We do not include the seven exchange-traded funds in this table. We obtain the historical versions of the designated securities list from the SSE and SZSE. Column 1 repots the date on which a revised list comes into effect. Column 2 reports the number of stocks that on the effective day. Column 3 reports the number of addition events that each time the list is revised. An addition event is defined as a stock is added to the list and therefore can be sold short. Vice versa, column 4 reports the deletion events. Column 5 reports the stock exchanges of which the revised stocks belong to.

### Data sources and sample selection

We obtain the information about the designated securities list from the Shenzhen and Shanghai stock exchanges. Capital data are obtained from the RESSET Financial Research Database, including daily stock returns, trading volume, volatility and capitalization. In order to make a comprehensive comparison, 2121 traded stocks are included in the sample as the basis for overall market performance, covering the calendar date from 4 January 2010 to 29 March 2013 with 785 trading days. Besides, we define the effective addition event as the stock is traded span from 30 days before effective date to 30 days after that. The choice of 30 trading day’s window is determined by the event study methodology (Brown and Warner 1985; Boehmer et al. 2013), the empirical findings of Chinese stock market (Zhang et al. 2013) and the aims of this study focusing on the relative short period of the impact of short selling. We are left with 351 effective addition events without a single stock in ChiNext. Therefore, the ChiNext stocks are excluded in the evaluation of overall market performance. This sample selection procedure allows us to examine the impact of short selling regulations on intraday volatility and returns meticulously.

### Relative ranking methodology

Short selling regulations in Chinese stock market are unique in that only stocks on a list of securities announced by stock exchanges can be sold short. According to the Shenzhen and Shanghai stock exchanges, the target securities are prudently determined considering the market capitalization, price exchange ratio and daily turnover. Therefore, it is impossible to find out comparative stocks in light of market situation and their own condition. For example, the initial 90 stocks added to the designate list on 31 March 2010 are the composite stocks of SSE 50 and SZSE 40, which are the largest stocks in both Shanghai Stock Exchange and Shenzhen Stock Exchange. Hence, any control group would bias the results to some extent. Moreover, as we all know that the individual stock performance is closely related to the overall performance of Chinese stock market. Macroeconomic environment play a key role in affecting the volatility of stock market (Engle and Rangel 2008), i.e., the interest rate, inflation rate and GDP can either exacerbate or decrease the stock volatility. Since the short selling regulations for different stocks are released at different time in different exchanges. It is apparently inappropriate for us to focus on the absolute volatility before and after stocks are included in the list because of the changing macroeconomic environment. For these two reasons, a relative ranking methodology is proposed to “difference out” the confounding factors of other concurrent events and isolate the short selling regulations effect.

To empirically investigate the effect of the implementation short selling regulations, we define the relative ranking score (RRS) as the relative position of a certain stock compared to the overall market performance. From this definition, we can compute the relative ranking score of volatility (RRSV), the relative ranking score of return (RRSR) and the relative ranking score of trading volume (RRSTV). Therefore, the lower of the RRSV is, the better of the implementation effect is.

1 $$RRSV = \frac{No.\;of\;Stocks\;with\;Higher\;Volatility\;than\;the\;Addition\;Event}{No.\;of\;Traded\;Stocks\;in\;the\;Market}$$

2 $$RRSR = \frac{No.\;of\;Stocks\;with\;Higher\;Returns\;than\;the\;Addition\;Event}{No.\;of\;Traded\;Stocks\;in\;the\;Market}$$

3 $$RRSTV = \frac{No.\;of\;Stocks\;with\;Higher\;Trading\;Volume\;than\;the\;Addition\;Event}{No.\;of\;Traded\;Stocks\;in\;the\;Market}$$

## Results and discussion

**Fig. 1:**
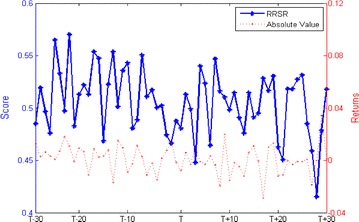
RRSR and its absolute value

**Fig. 2:**
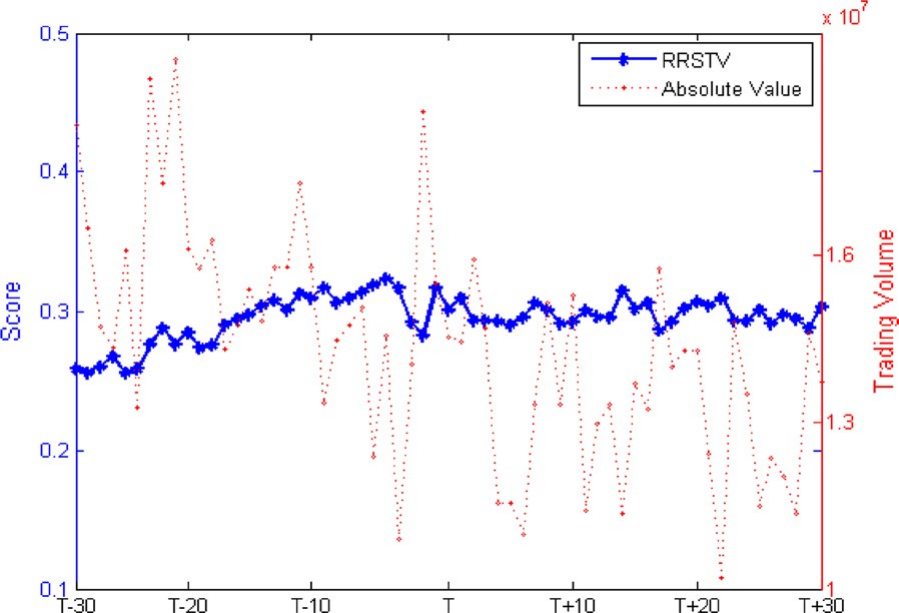
RRSTV and its absolute value

**Fig. 3:**
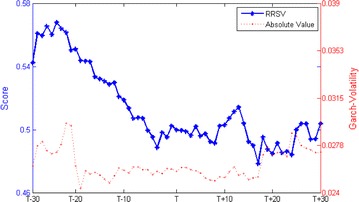
RRSV and its absolute value

We calculate the relative ranking scores and the absolute value of returns, trading volume and volatility during the estimation window of [−30, +30] trading days. Figure 1 plots the RRSR and its absolute value. Statistics data in Table 2 reports that both the RRSR (t = 1.9112) and the absolute value of returns (t = 1.7223) are insignificant. Figure 2 plots the RRSTV and its absolute value. Calculated from Table 2, we can infer that the trading activity is increase d by 14.89 % with statistically significant at the 1 % level (t = 7.0037) and the RRSTV is insignificant (t = −1.6735). Figure 3 plots the RRSV and its absolute value. Reported from Table 2, we find no discrepancies in the averages of absolute value between pre-event and post-event (t = −0.2241). However, the RRSV is significant increased by 6.463 % (t = 7.6690). Overall, these results show that short sellers in China destabilize the market, which is in accord with the Hong Kong evidence (Chang et al. 2007) and inconsistent with the international evidence (Saffi and Sigurdsson 2011). This happens because the diverse information is aggregated in the market when short selling regulations are implemented. Previous unfavorable information is reflected and resulted in overvaluation of prices. Our finding also confirms the conjecture by Allen and Gale (1991).

**Table 2:** Changes in RRS and its absolute value around effective addition events

	Pre-event	Post-event	t test
Panel A: RRS			
RRSR	0.5126	0.4994	1.9112
RRSTV	0.2913	0.2979	−1.6735
RRSV	0.5307	0.4964	7.6690***
Panel B: Absolute value			
Returns	0.0031	−0.00075	1.7223
Trading volume	1.5598 × 10^7^	1.3276 × 10^7^	7.0037***
Garch-volatility	0.0263	0.0263	−0.2241

*** Significance at the 1 % level

This table reports the changes around effective addition events. Column “Pre-event” shows the cross-sectional average of variables during the previous estimation window of [−30, −1] trading days relative to the effective addition events, and the “Post-event” shows the average in the post estimation window of [1, 30] trading days. Column “t test” shows the results of the paired t test to examine the statistical significance of the changes in average around the effective addition events.

**Fig. 4:**
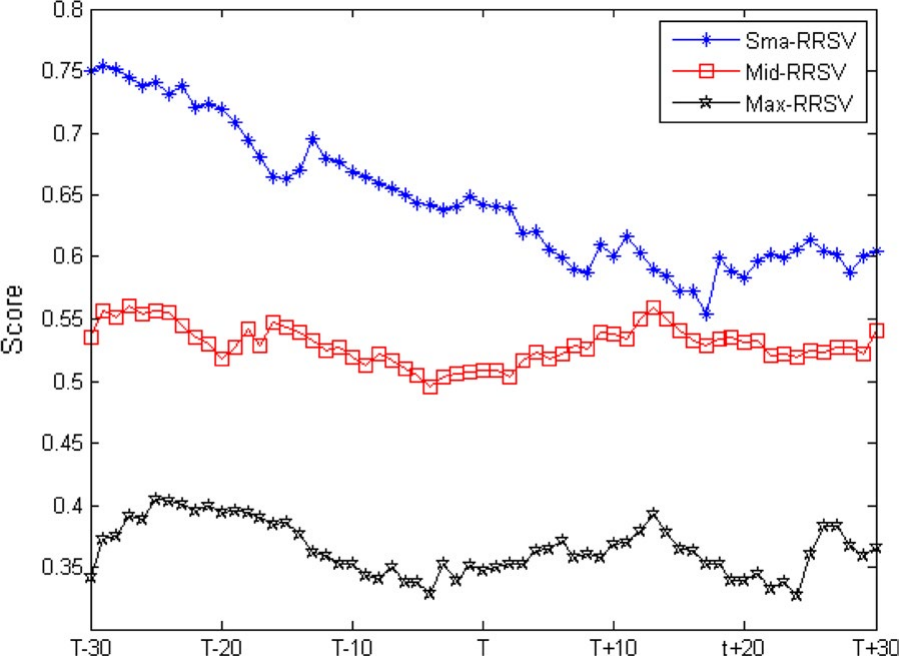
RRSV around the effective addition events based on capitalization

To better understand the implementation effect, we classify the effective addition events into three categories based on event day capitalization (Boehmer et al. 2013). Figure 4 shows that the small-cap stocks experience the sharpest increase in relative volatility (t = 14.4214). In the small-cap of effective addition events, the average relative ranking scores of volatility are 0.6918 and 0.5998 in the previous and post period. As calculated from Table 3, the relative volatility is increased by 13.30 %. In contract, the increase in relative volatility of middle-cap and maximum-cap stocks is less significant. These results reveal that the overall effect of the implementation of short selling mechanism in China is obvious for the small-cap stocks. In addition to the aggregate information function of the short sellers mentioned previous, the discrepancies among different capitalization categories maybe explained by the composition of the short sellers (informed traders or predatory traders) and the trading activity (Huang and Masulis 2003).

**Table 3:** Changes in RRS around effective addition events based on capitalization

	Pre-event	Post-event	t test
Sma-RRSV	0.6918	0.5998	14.4214***
Mid-RRSV	0.5303	0.5294	0.2332
Max-RRSV	0.3701	0.3599	2.3576**

** Significance at the 5 % level

*** Significance at the 1 % level

This figure plots the RRSV around the effective addition events based on the event day capitalization. Each category consists of 117 effective addition events.

**Fig. 5:**
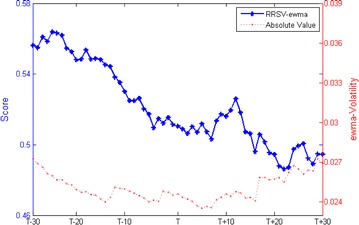
RRSV-ewma and its absolute value

**Fig. 6:**
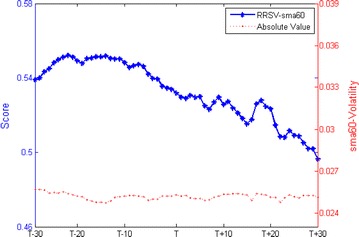
RRSV-sma60 and its absolute value

Our key finding is that short selling regulations facilitate the increase of intraday volatility relative to the overall market performance even the absolute value is almost invariant. Because it is impossible to find the ideal control group, we prudently confirm our results by employing two alternative calculations of volatility, i.e., the volatility of exponential weighted moving average (ewma-Volatility) and the volatility of 60 days simple moving average (sma60-Volatility). Figures 5 and 6 plot these two measures of volatility respectively. Table 4 report that there are no discrepancies in the averages of absolute value between pre-event and post-event but volatility relative to the overall market perform is statistical significant increased (t = 15.8478 and t = 18.1501). These results validate the main findings in this paper.

**Table 4:** Changes in RRS and its absolute value of ewma-Volatility and sma60-Volatility around effective addition events

	Pre-event	Post-event	t test
Panel A: RRS			
RRSV-ewma	0.5397	0.5033	15.8478***
RRSV-sma60	0.5461	0.5190	18.1501***
Panel B: Absolute value			
ewma-volatility	0.0250	0.0251	−0.3186
Sma60-volatility	0.0252	0.0252	0.1468

*** Significance at the 1 % level

## Conclusions

In this paper, we examine the impact of short selling regulations on Chinese market. We identify the effective addition events as the stocks are included to the designated securities list. We find these events are associated with statistical significant post-event increase in volatility relative to the overall market and absolute value of trading volume. Furthermore, we classify the events into three categories based on capitalization and the results suggest that small-cap stocks experience the sharpest increase in volatility relative to the overall market performance. Various calculations of volatility are also performed to test the robustness. These findings should be insightful to policy makers in China who have an interest in understanding the impact of short selling regulations on improving trading mechanism and boosting the develop of the market.
